# Follow-up study of depressive state on patients with atrial fibrillation 1 year after radio-frequency ablation

**DOI:** 10.3389/fpsyt.2022.1046924

**Published:** 2022-12-22

**Authors:** Lei Ren, Wenjun Li, Xin Su, Yangyang Yang, Yuanzhuo Zhang, Xiaozhu Liu, Guangquan Hu, Bin Ning

**Affiliations:** ^1^Department of Cardiovascular Medicine, Fuyang People’s Hospital Affiliated to Anhui Medical University, Fuyang, China; ^2^Department of Cardiovascular Medicine, Fuyang Hospital Affiliated to Bengbu Medical College, Fuyang, China; ^3^Department of Cardiovascular Medicine, The Second Affiliated Hospital of Chongqing Medical University, Chongqing, China; ^4^Department of Cardiovascular Medicine, The Second Affiliated Hospital of Anhui Medical University, Hefei, China

**Keywords:** SDS, depression, AF, radio frequency ablation, recurrence

## Abstract

**Objective:**

To analyze the effect of depression on the recurrence of atrial fibrillation (AF) 1 year after radio-frequency ablation.

**Methods:**

A total of 91 patients with AF admitted to our hospital from January 2020 to July 2021 were studied. All patients were followed up 1 year after radio-frequency ablation. A total of 91 subjects were divided into recurrence group (*n* = 30) and no recurrence group (*n* = 61) according to the recurrence situation 1 year after radio-frequency ablation. Age, disease course, body mass index (BMI), gender, echocardiography (left atrial diameter), blood inflammatory indicators (neutrophil count, lymphocyte count, and monocyte count), and Self-rating Depression Scale (SDS) scores were compared between the two groups. Logistic multivariate regression analysis was used to analyze the effect of SDS score and other indexes on the recurrence of AF 1 year after radio-frequency ablation.

**Results:**

The age of patients in relapse group was higher than that in no relapse group (*P* < 0.05) and the course of disease was longer than that of the no recurrence group (*P* < 0.05). The BMI was higher than that of the no recurrence group (*P* < 0.05) and the left atrial diameter was greater than that of the no recurrence group (*P* < 0.05). Neutrophil count and monocyte count were significantly higher than those in no recurrence group (*P* < 0.05) and the lymphocyte count was significantly lower than that in the no recurrence group (*P* < 0.05). There were significant differences in SDS score composition between the two groups (*P* < 0.05) and the composition ratio of patients with moderate and major depression in the relapsing group was significantly higher than that in the non-relapsing group. The composition ratio of patients without depression in the relapsing group was significantly lower than that in the non-relapsing group. Multivariate analysis showed that age, disease course, BMI, left atrial diameter, neutrophil count, lymphocyte count, monocyte count, and SDS score were all independent factors affecting the recurrence of AF patients 1 year after radio frequency ablation (*P* < 0.05). Compared with patients without depression, patients with mild, moderate and major depression had an increased risk of recurrence by 74.0, 98.2, and 151.2% 1 year after radio-frequency ablation, respectively (*OR* = 1.740, 1.982, and 2.512).

**Conclusion:**

There is a high rate of depression in patients with AF. Depression is an important factor affecting the early recurrence of patients with AF after radio-frequency ablation.

## 1 Introduction

Atrial fibrillation (AF) is one of the most common heart rhythm disorders in clinical practice. The electrophysiology is manifested as disordered atrial electrical activity. According to the characteristics of AF, it can be divided into paroxysmal AF, persistent AF, long-term persistent AF and permanent AF (1). Studies have shown that the morbidity of AF in the general population is 1–2% and the overall morbidity of AF in China is 0.77% (2). Studies have shown that the risk of stroke, myocardial infarction and other serious complications in patients with AF is four times than that of the normal population and AF is also one of the risk factors for cognitive dysfunction, which will lead to cognitive decline in patients (3). At present, the main treatment strategies for AF are controlling the heart rhythm through drugs, radio-frequency ablation, surgery and other ways to restore, and maintain the sinus rhythm of patients. However, the incidence of adverse reactions to drug therapy is high. Meanwhile, bleeding and other related risk events are easy to occur. Nevertheless, surgical wounds are always large, which, can cause obvious stress stimulation to patients, as well as other disadvantages such as long post-operative recovery time and adverse prognosis (4). Radio-frequency ablation has significant advantages in improving the quality of life and post-operative survival rate of patients with AF, and is the recommended AF treatment plan by the European Society of Cardiology and the European Surgical Association in 2020 (5). In a 5-year follow-up study, CABANA confirmed that compared with drug therapy, transcatheter radio-frequency ablation was more effectively reduce the recurrence rate of AF, especially the occurrence of symptomatic AF (6). However, in the group of patients with AF undergoing radio-frequency ablation for the first time, a part of them still relapsed within 3 months to 1 year after radio-frequency ablation and the early recurrence and high recurrence rate are also one of the difficulties in current research (7). Therefore, it is important to clarify the risk factors and mechanisms of early recurrence, which, could provide appropriate reference for early clinical intervention and it is also one of the important ways to reduce the risk of post-operative recurrence in patients with AF. Studies have shown that patients with AF complicated with depression and other adverse mental states are relatively common. Medical staff should pay attention to the mental health status of these patients during perioperative period and provide appropriate intervention when necessary (8). Because AF can repeatedly present paroxysmal palpitations and other clinical manifestations, both patients with AF and their accompanying family members may have a high incidence of depression after a long time of accompanying (9). In recent years, studies on AF and depression have become a research hotspot in this field. Patients with AF with depression and other adverse mental health conditions have more obvious clinical symptoms, higher severity, recurrence rate, and mortality of this disease (10). Whereas most of these studies are cross-sectional investigations. This study followed up 91 patients with AF in the first year after radio-frequency ablation, aiming to explore the influence of mental health status on post-operative recurrence and provide a basis for exploring effective intervention programs to reduce early post-operative recurrence from a psychological perspective.

### 1.1 Objects and methods

#### 1.1.1 Research objects

The study’s subjects were 91 patients with AF admitted to our hospital from January 2020 to July 2021. There were 50 males and 41 females; The age ranged from 31 to 83 years old, with a mean of (60.5 ± 15.3) years old. The course of disease was 1–5 years with an average of (2.0 ± 1.6) years.

Diagnostic criteria: It meets the diagnostic criteria for AF in the “Guidelines for the Diagnosis and Management of Atrial Fibrillation Developed by ESC and European Association of Cardiothoracic Surgery” in 2020 (11).

Inclusion criteria: diagnosed as paroxysmal AF; Radio-frequency ablation for the first time; >18 years old; NYHA grade I–III for preoperative cardiac function; Clear consciousness, normal cognitive level, and barrier-free communication; Volunteer to participate in this study.

Exclusion criteria: History of surgery or other neurological diseases within the last 3 months; Complicated with severe diseases such as tumor and vital organ failure; A history of mental illness; Those who have joined other research projects during the study; Those who haven’t completed followed-up study 1 year after radio frequency ablation.

## 2 Materials and methods

### 2.1 Collecting data

All enrolled patients underwent circum-pulmonary vein isolation guided by CARTO3 and were followed up for 1 year after surgery. All patients underwent imaging with GE vivid E95 ultrasound diagnostic apparatus (GE Healthcare), M5S probe (frequency: 1.5∼4.6 MHz), and 4 V probe (frequency: 1.5∼4.0 MHz) to complete the detection of the left atrium (LA). According to the recurrence situation 1 year after radio-frequency ablation, 91 subjects were divided into recurrence group (*n* = 30) and no recurrence group (*n* = 61). Clinical data of each group were collected: age, disease course, body mass index (BMI), gender, heart color doppler ultrasound (left atrial diameter), blood inflammatory indicators (neutrophil count, lymphocyte count, and monocyte count), and SDS score.

Self-rating Depression Scale (12) consisted of 20 items, depression severity index = accumulated score/80. A depression severity index below 0.50 indicates no depression, 0.50–0.59 indicates slight to mild depression, 0.60–0.69 indicates moderate to major depression and depression severity index above 0.70 indicates major depression.

### 2.1.1 Analysis of influencing factors for recurrence 1 year after surgery

Each item in the clinical data was set as the observation variables and univariate analysis was performed for the recurrence of AF patients 1 year after radio-frequency ablation. Univariate analysis was statistically significant (*P* < 0.05) was set as the independent variable and the recurrence 1 year after radio-frequency ablation was set as the dependent variable, which was jointly included in the logistic multivariate regression model for multivariate regression analysis.

### 2.2 Statistical analysis

SPSS 25.0 statistical software was used for data analysis. Normal measurement data were represented by ($\bar{x}\pm s$) and independent sample *t-*test was used to compare the mean of two samples. The statistical data were represented by the number of cases or percentage, the chi-square test was used for the comparison between the four groups and the non-parametric test was used for the comparison between the groups of the rank data. Logistic regression model was used for multivariate analysis. Hypothesis test level: α = 0.05.

## 3 Results

### 3.1 Comparison of baseline data between the two groups

According to the statistics in Table 1, the age of patients in the relapsing group was higher than that in the non-relapsing group (*P* < 0.05), the course of disease was longer than that of the no recurrence group (*P* < 0.05), the BMI was higher than that of the no recurrence group (*P* < 0.05), the left atrial diameter was greater than that of the no recurrence group (*P* < 0.05), neutrophil count and monocyte count were significantly higher than those in no recurrence group (*P* < 0.05), the lymphocyte count was significantly lower than that in the no recurrence group (*P* < 0.05).

**TABLE 1:** The comparison of baseline information between two groups.

Variables	Recurrent group (*n* = 30)	No-recurrent group (*n* = 61)	*t*/χ*^2^*	*P*-value
Age (years, $\bar{x}\pm s$)	62.51 ± 7.32	57.33 ± 7.54	3.110	0.003
Course of disease (years, $\bar{x}\pm s$)	3.56 ± 1.36	2.75 ± 1.47	2.601	0.011
BMI (kg/m*^2^*, $\bar{x}\pm s$)	25.11 ± 2.02	23.19 ± 2.54	3.613	<0.001
**Gender (*n*%)**			0.054	0.817
Male	17 (56.66)	33 (54.09)		
Female	13 (43.33)	28 (45.90)		
Inner diameter of left atrium (mm, $\bar{x}\pm s$)	43.05 ± 2.25	36.26 ± 2.36	13.098	<0.001
Neutrophil count (×10*^9^*/L, $\bar{x}\pm s$)	3.62 ± 0.56	2.91 ± 0.27	8.185	<0.001
Lymphocyte count (×10*^9^*/L, $\bar{x}\pm s$)	1.50 ± 0.23	1.70 ± 0.36	2.773	0.007
Monocyte count (×10*^9^*/L, $\bar{x}\pm s$)	0.39 ± 0.05	0.32 ± 0.04	7.214	<0.001

### 3.2 Comparison of SDS scores between the two groups

Non-parametric test results showed that there were significant differences in the composition of SDS scores between the two groups (*P* < 0.05). In general, the composition ratio of patients with moderate and major depression in the relapsing group was significantly higher than that in the non-relapsing group and the composition ratio of patients without depression in the relapsing group was significantly lower than that in the non-relapsing group. See Table 2.

**TABLE 2:** Comparison of SDS score between two groups (*n*%).

SDS score	Recurrent group (*n* = 30)	No recurrent group (*n* = 61)	*Z*-value	*P*-value
<0.5 (no depression)	13 (43.33)	41 (67.21)	−2.671	0.008
0.50∼0.59 (mild depression)	8 (26.66)	16 (26.22)		
0.60∼0.69 (moderate depression)	5 (16.66)	3 (4.91)		
>0.7 (major depression)	4 (13.33)	1 (1.63)		

### 3.3 Effects of SDS score and other indexes on recurrence 1 year after radio-frequency ablation

Self-rating depression scale score and other indicators (age, course of disease, BMI, left atrial diameter, neutrophil count, lymphocyte count, and monocyte count) were set as independent variables and the recurrence 1 year after radio-frequency ablation was set as dependent variable, which were then included in the logistic multivariate regression analysis of recurrence. The specific assignment scheme of each variable is shown in Table 3. Multivariate analysis showed that age, disease course, BMI, left atrial diameter, neutrophil count, lymphocyte count, monocyte count, and SDS score were all independent factors affecting the recurrence of AF patients 1 year after radio frequency ablation (*P* < 0.05).

**TABLE 3:** Each variable and assignment.

Variables	Meaning	Assignment
X1	Age	Actual value
X2	Course of disease	Actual value
X3	BMI	Actual value
X4	Inner diameter of left atrium	Actual value
X5	Neutrophil count	Actual value
X6	Lymphocyte count	Actual value
X7	Monocyte count	Actual value
X8	SDS score*	No depression: X8a = 0, X8b = 0, X8c = 0 Mild depression: X8a = 1, X8b = 0, X8c = 0 Moderate depression: X8a = 0, X8b = 1, X8c = 0 Major depression: X8a = 0, X8b = 0, X8c = 1
Y	1-year occurrence after radio frequency ablation	0 = No;1 = Yes

*Multiple classification variable, Dummy variables need to be set.

**FIGURE 1:**
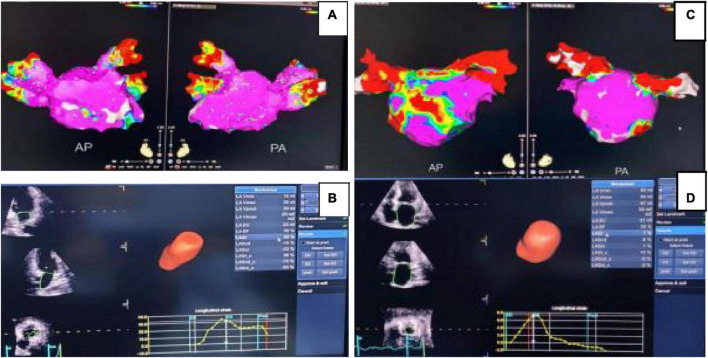
Forest plot of 1-year risk of relapse for the patients with AF after radio-frequency ablation. In panel **(A)**, the left atrial diameter is normal. Group electro-anatomical mapping sample diagram, purple represents non-low voltage region; **(B)** is the echocardiography of the same patient without recurrence. In panel **(C)**, the left atrial diameter of the relapsed group increased significantly and purple represents the non-low voltage region. **(D)** is the echocardiogram of the same patient in the relapse group.

As it can be seen from the specific analysis results in Table 4, the risk of recurrence 1 year after radio-frequency ablation will increase by 41.1% for each additional year of patient age (*OR* = 1.411). The risk of recurrence 1 year after radio-frequency ablation increased by 30.7% (*OR* = 1.307) for each additional year of the course of disease. For every 1 kg/m2 increase in BMI, the risk of recurrence 1 year after surgery increased by 56.7% (*OR* = 1.567). For every 1 mm increase in left atrial diameter, the risk of recurrence 1 year after surgery increased by 76.6% (*OR* = 1.766). For each increase of neutrophil count and monocyte count by 1 × 10^9^/L, the risk of recurrence 1 year after surgery increased by 40.4 and 45.5%, respectively (*OR* = 1.404 and 1.455). For every 1 × 10^9^/L increase in lymphocyte count, the risk of recurrence 1 year after surgery was reduced by 37.9% (*OR* = 0.621). Statistical data on SDS scores showed that patients with mild, moderate and major depression had an increased risk of recurrence 1 year after surgery of 74.0, 98.2, and 151.2%, respectively, compared with patients without depression (*OR* = 1.740, 1.982, and 2.512). It can be seen from the specific analysis results in Figure 1 that the presence of mild, moderate, and major depression as well as a higher left atrial diameter have a greater impact on the recurrence of AF patients 1 year after radio-frequency ablation.

**TABLE 4:** Effect of SDS score and other indicators on recurrence 1 year after radio-frequency ablation.

Variables	β	SE	Wald x^2^	*P*-value	OR	95% CI
Age	0.344	0.752	5.369	0.001	1.411	1.205∼5.332
Course of the disease	0.268	1.023	4.035	0.015	1.307	1.068∼4.061
BMI	0.449	1.047	6.332	<0.001	1.567	1.121∼4.652
Inner diameter of left atrium	0.569	0.677	8.566	<0.001	1.766	1.134∼6.788
Neutrophil count	0.339	0.511	6.337	<0.001	1.404	1.033∼6.884
Lymphocyte count	−0.477	0.692	7.153	<0.001	0.621	0.365∼3.412
Monocyte count	0.375	1.124	10.062	<0.001	1.455	1.266∼4.779
SDS score*			12.325	<0.001		
Mild depression	0.554	1.136	6.385	<0.001	1.740	1.233∼4.035
Moderate depression	0.684	1.031	7.422	<0.001	1.982	1.366∼4.661
Major depression	0.921	1.243	8.512	<0.001	2.512	1.439∼5.688
Term of constant	−3.448	0.564	12.314	<0.001	0.032	
						

## 4 Discussion

Studies on the relationship between the occurrence and severity of AF and the adverse mental health have been going on for a long time. Data have confirmed that anxiety, depression and other adverse mental states can increase the probability of occurrence of AF by 4–6 times (13). Depression and other adverse mental states are relatively common in patients with AF (14). The data in this study (Table 2) also showed that among the 91 patients with AF, 37 (40.7%) had depression before surgery, including 24 (26.37%) with mild depression, 8 (8.79%) with moderate depression, and 5 (5.49%) with severe depression. The high rate of depression in patients with AF may be due to the following reasons (15): First, many patients with AF have insufficient professional knowledge of the disease and regard AF as an end-stage heart disease and its related complications as an inevitable outcome, so they are under great psychological pressure. Secondly, patients with AF usually feel fatigue, chest tightness, and other symptoms, which, affect the quality of life, so patients with atrial fibrillation are prone to anxiety and depression. Finally, some patients worry too much about the side effects of drugs or surgical trauma, so they are often in a state of anxiety and depression.

In the previous literature study, the author found that there are many researches on physical factors in the field of early recurrence of AF after radio-frequency ablation, but the studies on early recurrence of AF after radio-frequency ablation and depression are still in the initial stage and there are few relevant reports. To summarize the conclusions of previous studies, the mechanism of occurrence or recurrence of AF caused by depression may involve the following aspects (16, 17): Anxiety and depression can activate the renin-angiotensin-aldosterone system, play a significant role in promoting cardiac fibrosis, delay atrial conduction and reentry, increase atrial pressure, change atrial electrophysiological remodeling, and thus lead to the occurrence or recurrence of atrial fibrillation. Anxiety and depression can lead to a high level of inflammatory factors and reactive oxygen species in the body, promote myocardial fibrosis and significantly affect the occurrence and recurrence of AF. Anxiety, depression and mania can cause significant increase in the level of catecholamines released by the body, which is also an important factor affecting the dysfunction of the autonomic nervous system of patients, providing conditions for the occurrence or recurrence of AF. In this study, the preoperative SDS scores of patients in the relapsing group and the non-relapsing group were compared, aiming to preliminarily analyze the relationship between the recurrence of atrial fibrillation patients 1 year after radio-frequency ablation and the depressive state of the patients. The results showed that both groups had a certain rate of depression, but the recurrence group was significantly more serious than the non-recurrence group. The composition of moderate and severe depression patients in the recurrence group was higher (16.66 and 13.33%), while the composition of non-depression patients was lower (43.33%). The proportion of patients with mild depression was similar between the two groups (26.66 vs. 26.22%). These results indicate that the depression status of atrial fibrillation patients has certain influence on the early recurrence after radio-frequency ablation.

At present, although the pathophysiological mechanism of AF recurrence has not been fully elucidated, it has been also confirmed that inflammation and oxidative stress levels are closely related to the occurrence of atrial myocyte fibrosis in patients with AF (18). Neutrophils are the main markers of inflammation in the body and can reflect the subclinical inflammatory state of patients. Monocytes are important factors mediating inflammation and oxidative stress reaction processes. Lymphocyte levels correlate with the level of oxidative stress in the body. In addition, the ratio of neutrophils to lymphocytes (NLR) is one of the predictors of AF onset and long-term prognosis. The higher the NLR is, the higher the risk of AF and the worse the long-term prognosis (19, 20). In this study, it was found that the percentage of neutrophil in the relapsing group was significantly higher than that in the non-relapsing group and NLP was basically consistent with the above studies. It also provides a new reference scheme for the later clinical treatment of AF. The inner diameter of the left atrium can be obtained by cardiac color Doppler ultrasound detection, and is a clinically recognized indicator closely related to the recurrence of AF (21). Studies suggest that left atrial remodeling and left atrial volume increase are positively correlated with the occurrence of AF, which, are also one of the predictors of the recurrence of AF after circumferential pulmonary vein isolation (22–25). As shown in Figure 1, the patient with an enlarged left atrial diameter was found to have recurrent AF at a 1-year follow-up after radio-frequency ablation.

In order to further analyze the influence of psychological factors on the recurrence of AF within 1 year after radio-frequency ablation, SDS score and other commonly used and recognized indicators closely related to the recurrence of AF (inflammation indicators, left atrial diameter, etc.) were included in the multivariate regression analysis of post-operative recurrence in this study. The results showed that depressive state was an independent factor affecting the recurrence of atrial fibrillation patients at 1 year after radio-frequency ablation (*P* < 0.05). Compared with patients without depression, patients with mild, moderate and severe depression had an increased risk of recurrence 1 year after surgery of 74.0, 98.2, and 151.2%, respectively. Moreover, compared with other influencing factors, depression has a greater effect on the recurrence of AF patients 1 year after radio-frequency ablation. It is suggested that if patients with AF are complicated with depression, the risk of early post-operative recurrence will be significantly increased and the screening of anxiety and depression should be strengthened in the management of patients with AF.

In summary, this study found that there is a high risk of occurrence of depression in patients with AF and depression is an important factor affecting the early recurrence of patients with AF after radio-frequency ablation. This also suggests that medical staff should pay attention to the mental health status of patients in time, and take appropriate psychological intervention measures to improve the adverse mood of patients in order avoid the occurrence of adverse prognosis after AF. The sample size of this study is small and it is a single-center follow-up study, so the conclusions of this study have certain limitations. In the follow-up study, the number of samples and evaluation indicators will be further increased to explore the influence and mechanism of adverse mental health status on post-operative short-term and long-term recurrence from multiple perspectives.

## Data availability statement

The raw data supporting the conclusions of this article will be made available by the authors, without undue reservation.

## Author contributions

LR, BN, GH, and XL contributed to the conception and design. WL, XS, YY, and YZ collected and analyzed the data. LR, WL, and YY drew the figures, tables and wrote the draft. LR, YY, and BN contributed to manuscript writing and revision. All authors approved the final manuscript.
